# Differential effects of vilazodone versus citalopram and paroxetine on sexual behaviors and serotonin transporter and receptors in male rats

**DOI:** 10.1007/s00213-015-4198-1

**Published:** 2016-01-13

**Authors:** Ronald S. Oosting, Johnny S. Chan, Berend Olivier, Pradeep Banerjee, Yong Kee Choi, Frank Tarazi

**Affiliations:** Division of Pharmacology, Utrecht Institute for Pharmaceutical Sciences, Utrecht University, Universiteitsweg 99, 3584 CG Utrecht, The Netherlands; Department of Psychiatry, Yale University School of Medicine, New Haven, CT USA; Forest Research Institute, Jersey City, NJ USA; Department of Psychiatry and Neuroscience Harvard Medical School and McLean Hospital, Belmont, MA USA

**Keywords:** Sexual behavior, 5-HT_1A_, 5-HT_2A_, 5-HT_T_, 5-HT, Vilazodone, Paroxetine, Citalopram, Ejaculatory behavior, SSRI

## Abstract

**Rationale:**

Sexual side effects are commonly associated with selective serotonin reuptake inhibitor (SSRI) treatment. Some evidence suggest that activation of 5-HT_1A_ receptors attenuates SSRI-induced sexual dysfunction.

**Objective:**

This study in male rats compared the effects of vilazodone, an antidepressant with SSRI and 5-HT_1A_ receptor partial agonist activity, with other prototypical SSRIs (citalopram and paroxetine) on sexual behaviors and 5-HT receptors (5-HT_1A_ and 5-HT_2A_) and transporter (5-HT_T_) levels in select forebrain regions of the limbic system using quantitative autoradiography.

**Methods:**

Rats received vilazodone (1, 3, and 10 mg/kg), citalopram (10 and 30 mg/kg), or paroxetine (10 mg/kg) treatment for 14 days. Sexual behaviors (frequency and latency of mounts, intromissions, and ejaculations) were measured in the presence of an estrous female rat on days 1 (acute), 7 (subchronic), and 14 (chronic).

**Results:**

Vilazodone-treated rats exhibited no sexual dysfunction compared with controls; in contrast, the citalopram- and paroxetine-treated rats exhibited impaired copulatory and ejaculatory behaviors after subchronic and chronic treatments. Chronic vilazodone treatment markedly decreased 5-HT_1A_ receptor levels in cortical and hippocampal regions, while the SSRIs increased levels of this receptor in similar regions. All chronic treatments reduced 5-HT_T_ levels across the forebrain; however, the magnitude of the decrease was considerably smaller for vilazodone than for the SSRIs.

**Conclusions:**

The current studies showed that chronic treatment with vilazodone, in contrast to citalopram and paroxetine, was not associated with diminished sexual behaviors in male rats, which may be related to the differential effects of vilazodone on 5-HT_1A_ receptor and 5-HT_T_ levels relative to conventional SSRIs.

## Introduction

Major depressive disorder (MDD) is one of the most common mental disorders, with an estimated lifetime prevalence in the USA of 19.2 % (Kessler et al. 2010). Selective serotonin reuptake inhibitors (SSRIs) are efficacious in treating MDD and are the most commonly prescribed first-line therapy. However, their clinical effectiveness is often limited by poor adherence and discontinuation due to lack of therapeutic response and adverse effects (Nantz et al. 2009). Sexual dysfunction, which affects up to 60 % of patients treated with SSRIs (Kennedy and Rizvi 2009), is a leading cause of treatment nonadherence (Ashton et al. 2005) and is reported by patients to be one of the most unacceptable side effects associated with SSRIs (Hu et al. 2004). Therefore, reducing the incidence and burden of sexual dysfunction associated with SSRIs is important in improving clinical outcomes in patients with MDD.

Approaches to mitigating the sexual dysfunction associated with SSRI treatment include lowering dosage, switching antidepressant medications, or adding concomitant medications such as a phosphodiesterase inhibitor type 5 (PDE5; e.g., sildenafil), a norepinephrine-dopamine reuptake inhibitor (e.g., bupropion), or a 5-HT_1A_ receptor partial agonist (e.g., buspirone) (Rizvi and Kennedy 2013). Clinical and preclinical evidence supports a role for the 5-HT_1A_ receptor in regulating sexual behavior. Buspirone was shown to improve sexual function in depressed patients that were experiencing sexual dysfunction while taking the SSRIs paroxetine and citalopram (Landen et al. 1999). In male rats, buspirone and full 5-HT_1A_ receptor agonists (8-OH-DPAT and flesinoxan) promoted sexual behavior by decreasing ejaculation latencies (Chan et al. 2010; Snoeren et al. 2014). In contrast, the 5-HT_1A_ receptor antagonist WAY-100635 inhibited sexual behavior when it was coadministered with citalopram (de Jong et al. 2005a) or paroxetine (Looney et al. 2005) and when administered in 5-HT_T_ knockout rats (Chan et al. 2011). These studies suggest that 5-HT_1A_ receptor antagonism only inhibits sexual behaviors under conditions of chronically elevated serotonin (5-HT) (Olivier et al. 2011).

Vilazodone is an SSRI and 5-HT_1A_ receptor partial agonist (Dawson and Watson 2009) approved by the Food and Drug Administration (FDA) for the treatment of MDD in adults (Forest 2011). Efficacy for vilazodone in adults with MDD was demonstrated in two pivotal trials (Khan et al. 2011; Rickels et al. 2009); safety was further supported in a 52-week open-label study (Robinson et al. 2011). Vilazodone treatment was associated with relatively low adverse impact on sexual function relative to the high prevalence of sexual dysfunction in patients at baseline (Clayton et al. 2013). Limited evidence from these clinical trials in MDD has shown that the incidence of sexual dysfunction was similar between patients treated with vilazodone and patients treated with placebo, suggesting that vilazodone may be associated with less sexual dysfunction than other SSRIs (Clayton et al. 2013).

The mechanism by which 5-HT_1A_ receptor agonists affect sexual behavior is not well understood. Previous evidence showing that 5-HT_1A_ receptor agonists can stimulate male sexual function (Blier and Ward 2003; Chan et al. 2010; Snoeren et al. 2014) suggests that the clinical advantages of vilazodone may in part be mediated through its activity at the 5-HT_1A_ receptor. This study compared the effects of vilazodone treatment and other SSRIs on copulatory and ejaculatory behaviors, as well as on brain 5-HT receptor and transporter levels in select forebrain regions of the limbic system in male rats.

## Materials and methods

### Animals

Male and female Wistar rats (Charles River Laboratories, Germany), initially weighing 250-300 g, were group housed (4 per cage) and maintained under reversed 12/12 h day/night cycles (lights off 7:00 a.m.; lights on 7:00 p.m.); food and water were available ad libitum. Estrus was induced in female rats by a single injection of 50 μg estradiol benzoate dissolved in sesame oil 36 to 42 h before testing with a male rat. Animals were weighed on days 1, 7, and 14 of the drug experiment. All behavioral experiments, including the drug administrations, were reviewed and approved by Utrecht University’s animal welfare committee (DEC).

### Drugs

Vilazodone hydrochloride (1, 3, 10 mg/kg) and citalopram hydrochloride (10, 30 mg/kg) were received from Forest Laboratories, LLC. Paroxetine (10 mg/kg) was obtained from a local pharmacy (20 mg tablet, Hexal Pharma Nederland BV) and crushed into powder form. All drugs were dissolved or suspended in vehicle (1 % methylcellulose and water) and were administered orally (PO) at a dose volume of 2 mL/kg.

### Sexual behavior test

The sexual behavior test was performed as previously described (Chan et al. 2010). Briefly, estrus was induced in female rats with estradiol injection 36 to 42 h before testing. Male rats were placed in an observation cage (30 × 40 × 60 cm) for a 30-min habituation period. Following the habituation period, an estrous female was placed in the cage and video recording commenced for 30 min. The frequencies of mounts (no vaginal penetration), intromissions (vaginal penetration), and ejaculations, and the latency to the first ejaculation (time between first mount to ejaculation) were measured and scored during the 30-min test using Observer® 5.0 (Noldus, Wageningen, The Netherlands). Copulatory efficiency was defined as [number of intromissions / (number of intromissions + number of mounts)] × 100 %. Data from the first ejaculation series, which included all events occurring before the first ejaculation, were reported for mounting frequency, ejaculation latency, and copulatory efficiency. The ejaculation frequency for the entire 30-min test was reported. All assessments were performed in the dark phase of the light/dark cycle under dim red light conditions.

### Sexual training and the selection of male rats

In order to achieve stable sexual behavior, male rats (*N* = 145) were trained once per week for five consecutive weeks (pretreatment) against an estrous female in an observation cage. Male rats that exhibited an ejaculation frequency of 2–3 per 30-min test in the final two of the five training sessions were classified as normal-performers (Pattij et al. 2005) and were included in the drug studies (*n* = 98).

### Experimental design

#### Sexual behavior test

Normal-performing male rats were randomly divided into seven treatment groups (*n* = 14/group): vehicle, vilazodone 1 mg/kg, vilazodone 3 mg/kg, vilazodone 10 mg/kg, citalopram 10 mg/kg, citalopram 30 mg/kg, and paroxetine 10 mg/kg. All treatments were administered once daily for 14 days, either 1 h before the sexual test or around 11:00 a.m. on days without a sex test. The evaluations of sexual behaviors were performed on day 1 (acute), day 7 (subchronic), and day 14 (chronic).

#### Animal treatment and tissue preparation

Following the chronic (14 days) treatment with vilazodone (3, 10 mg/kg), citalopram (10 mg/kg), paroxetine (10 mg/kg), or vehicle (1 % methylcellulose and water) and the day 14 sexual behavior tests, *n* = 8 animals per treatment group were randomly chosen for the autoradiography experiments. Rats were decapitated, and their brains were rapidly excised and stored at −80 °C. Next, coronal sections (10 μm) were cut on a cryostat at −20 °C and stored at −80 °C. Brain tissue from regions that are thought to mediate neural pathways and behaviors (and are typically disturbed in MDD patients) were selected for evaluation (Baldessarini 2006). The following brain regions selected for quantitative autoradiography: medial prefrontal cortex (MPC), dorsolateral frontal cortex (DFC), medial caudate putamen (CP-M), lateral caudate putamen (CP-L), nucleus accumbens (NAc), hippocampus CA_1_ region (HIPP-CA_1_), hippocampus CA_3_ region (HIPP-CA_3_), and entorhinal cortex (EC).

#### Autoradiography assays

Serotonin transporter (5-HT_T_) levels were determined as previously described (Zhang et al. 2002). First, sections were preincubated for 2 h at 4 °C in 50 mM Tris-HCl buffer (pH 7.4) containing 120 mM NaCl. Next, sections were incubated for 24 h at room temperature (RT) with fresh buffer containing 1 nM [^3^H]cyanoimipramine (5-HT_T_ ligand) and the nonspecific binding (NSB) agent 5 mM sertraline. Following incubation, sections were washed twice for 30 min in ice-cold buffer, rinsed in water, and air dried.

5-HT_1A_ receptor levels were determined as previously described (Tarazi et al. 2002). Sections were preincubated for 60 min at RT in 50 mM Tris-HCl buffer (pH 7.6) containing ascorbic acid (0.1 %, *w*/*v*), 4 mM CaCl_2_, and 10 μM pargyline-HCl. Next, sections were incubated for 1 h at RT with fresh buffer containing 2.0 nM [^3^H]8-OH-DPAT and 1 μM 5-HT as the NSB agent. Following incubation, sections were washed twice for 5 min in ice-cold buffer, rinsed in water, and air dried.

5-HT_2A_ receptor levels were determined as previously described (Tarazi et al. 2002). Sections were preincubated for 1 h at RT in 50 mM Tris-HCl buffer (pH 7.7). Next, sections were incubated for 1 h at RT in fresh buffer containing 3.0 nM [^3^H]ketanserin, 1 μM prazosin, and 100 nM tetrabenzine with the NSB 1 μM methysergide. Following the incubation, sections were washed twice for 30 min in ice-cold buffer, rinsed in water, and air-dried.

For image analysis, radiolabeled slides and calibrated [^3^H]standards (Amersham) were exposed to Biomax MR films for 5–6 weeks at 4 °C. The films were developed and fixed in Kodak D-19 for 5 min at RT. Optical density (OD) in brain regions of interest was measured with a computerized densitometric image analyzer (MCID-4 system, Imaging Research; St. Catharines, Ontario). OD was converted to nCi/mg of tissue with calibrated [^3^H]standards. After subtracting nonspecific binding from total binding, the specific binding was determined and expressed as femtomole bound per milligram tissue.

### Statistical analysis

All behavioral data were analyzed by analysis of variance (ANOVA; SPSSv22.0) to test for overall significance. If overall significant effects were found, post hoc Dunnett *t* tests were used to evaluate differences between active- and vehicle-treatment groups on the same experimental day. Data from the autoradiography assays were analyzed using a two-way ANOVA to evaluate changes across treatments and brain regions for each assay. In case of overall statistically significant effects for drug or brain region, post hoc Dunnett *t* tests were used to evaluate differences between treatment and vehicle groups. All significance testing was two-sided at the *P* < .05 level. All values are shown as the mean and standard error of the mean (SEM).

## Results

### Sexual behavior tests

#### Day 1/acute administration

**Fig. 1:**
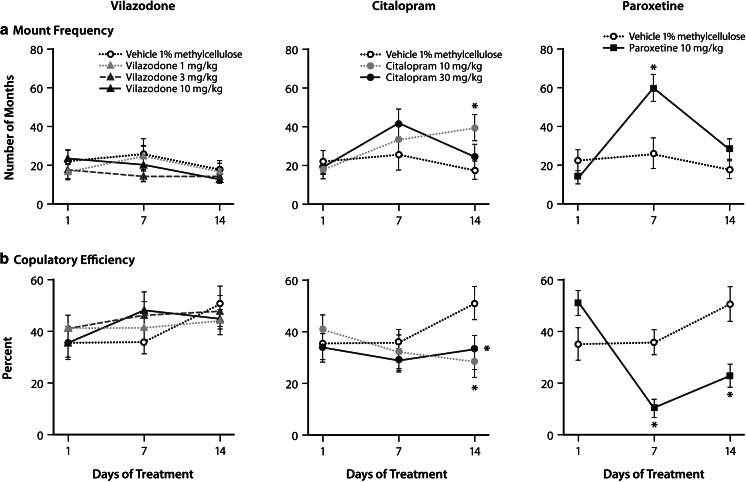
Effects of vilazodone, citalopram, and paroxetine on first series copulatory behavior in male rats. Effects of vilazodone (1, 3, and 10 mg/kg), citalopram (10 and 30 mg/kg), and paroxetine (10 mg/kg) on **a** mount frequency and **b** copulatory efficiency (defined as number of intromissions / [number of intromissions + number of mounts]) × 100 % during the first ejaculatory series of the 30-min sex conducted after treatment on day 1 (acute), day 7 (subchronic), and day 14 (chronic) in male Wistar rats (*n* = 14/drug/dose). Data are shown as the mean ± SEM. *P < .05 compared with vehicle

**Fig. 2:**
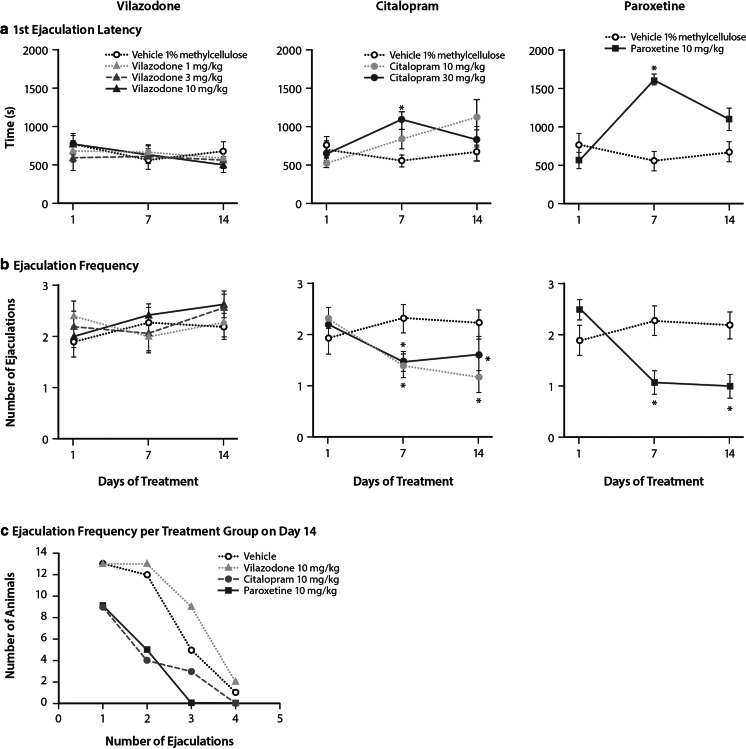
Effects of vilazodone, citalopram, and paroxetine on ejaculatory behavior in male rats. Effects of vilazodone (1, 3, and 10 mg/kg), citalopram (10 and 30 mg/kg), and paroxetine (10 mg/kg) on **a** first ejaculation latency and **b** total ejaculation frequency during the 30-min sex tests conducted after treatment on day 1 (acute), day 7 (subchronic), and day 14 (chronic) in male Wistar rats (*n* = 14/drug/dose). Data are shown as the mean ± SEM. *P < .05 compared with vehicle. **c** The number of rats (*n* = 14/group) that had 1, 2, 3, or 4 ejaculations during the 30-min sexual behavior test on day 14

On day 1, the vehicle-treated rats displayed an average of 2.5 ejaculations. Following acute administrations of vilazodone, citalopram, and paroxetine, there were no significant effects of treatment on any of the sexual behavior parameters (Figs. 1 and 2a–b)

#### Day 7/subchronic administration

There were no differences in vehicle-treated rats on day 7 compared with day 1, indicating stable endophenotypes of the sexual behavior parameters.

For the day 7 tests, there were significant effects of treatment on mount frequency (*F*_6, 91_ = 5.52; *P* < .001), copulatory efficiency (*F*_6, 91_ = 5.61; *P* < .001), latency to first ejaculation (*F*_6, 91_ = 12.65; *P* < .001), and ejaculation frequency (*F*_6, 91_ = 3.94; *P* = .002). Post hoc comparisons showed that vilazodone-treated rats did not display any significant differences from vehicle-treated rats in sexual behavior after 7 days of treatment. In contrast, citalopram-treated rats displayed increased latency to ejaculation and decreased ejaculation frequency compared with vehicle-treated controls (*P* < .05) (Fig. 2a–b). Paroxetine-treated rats displayed significantly impaired sexual behavior on all measures after 7 days of treatment (*P* < .05) (Figs. 1 and 2).

#### Day 14/chronic administration

**Fig. 3:**
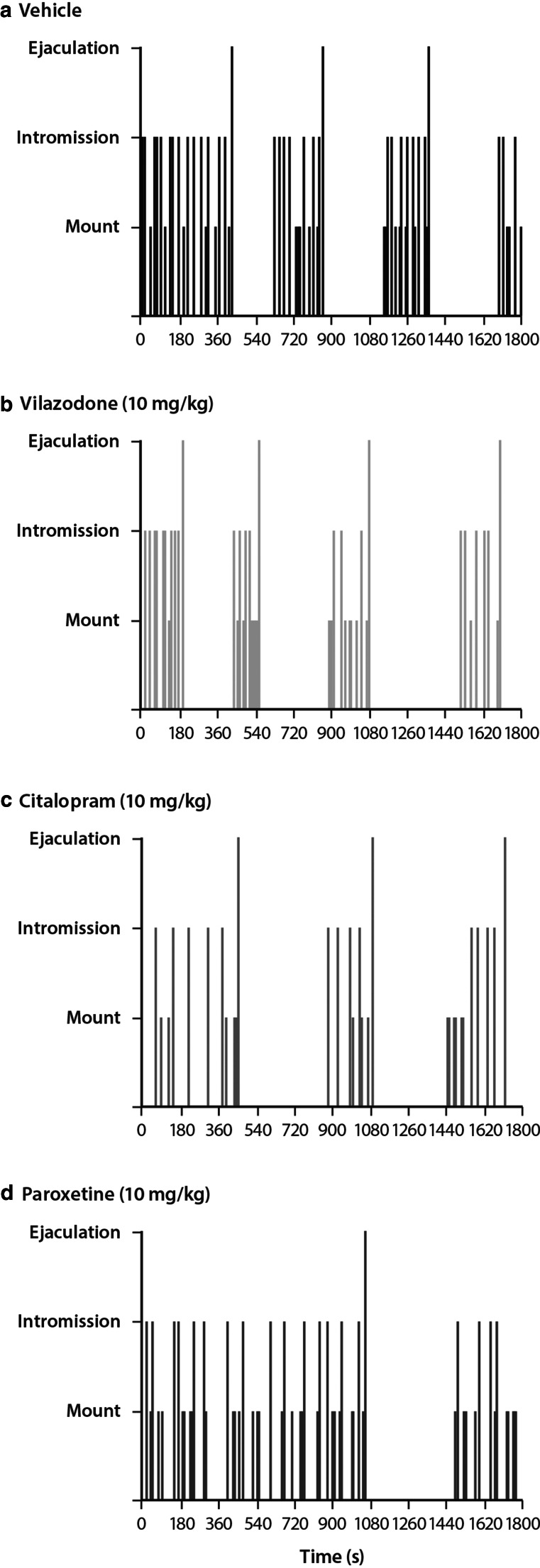
Representative traces of individual sexual function after chronic (14 days) treatment. Representative traces of sexual function during the 30-min sex test (x-axis) of individual rats (*n* = 1/group) treated with **a** vehicle, **b** vilazodone, **c** citalopram, or **d** paroxetine (10 mg/kg) on day 14. Y-axis shows the events scored during the test (number of mounts, intromissions, and ejaculations)

Representative traces of individual rat scores for mounts, intromissions, and ejaculations during the 30-min sex test on day 14 (i.e., chronic treatment) are shown in Fig. 3.

On day 14, there were significant effects of treatment for mount frequency (*F*_6, 91_ = 3.59; *P* = .003), copulatory efficiency (*F*_6, 91_ = 3.83; *P* = .002), latency to first ejaculation (*F*_6, 91_ = 4.10; *P* = .001), and ejaculation frequency (*F*_6, 91_ = 5.88; *P* < .001). Similar to the previous test days, vilazodone-treated rats did not display any differences in the sexual behaviors compared with vehicle-treated rats. In contrast, citalopram-treated rats had an increase in mount frequency (*P* < .05; 10 mg/kg) yet decreased ejaculation frequency and copulatory efficiency (*P* < .05; 10 and 30 mg/kg) (Figs. 1 and 2b). Paroxetine-treated rats displayed no differences in mount frequency and ejaculation latency compared to vehicle-treated rats; however, ejaculation frequency and copulatory efficiency were decreased (*P* < .05) (Figs. 1 and 2).

During the day 14 sex test, the number of rats exhibiting 1, 2, 3, or 4 ejaculations was comparable following chronic treatment with vilazodone (10 mg/kg; single dose tested) or vehicle, suggesting that chronic vilazodone treatment had no adverse effects on ejaculations. Chronic treatment with citalopram or paroxetine resulted in fewer rats having 1, 2, 3, or 4 ejaculations during the test than rats treated with vehicle or vilazodone, suggesting that these treatments adversely affected ejaculation frequency (Fig. 2c).

### 5-HT_1A_ receptor levels

**Fig. 4:**
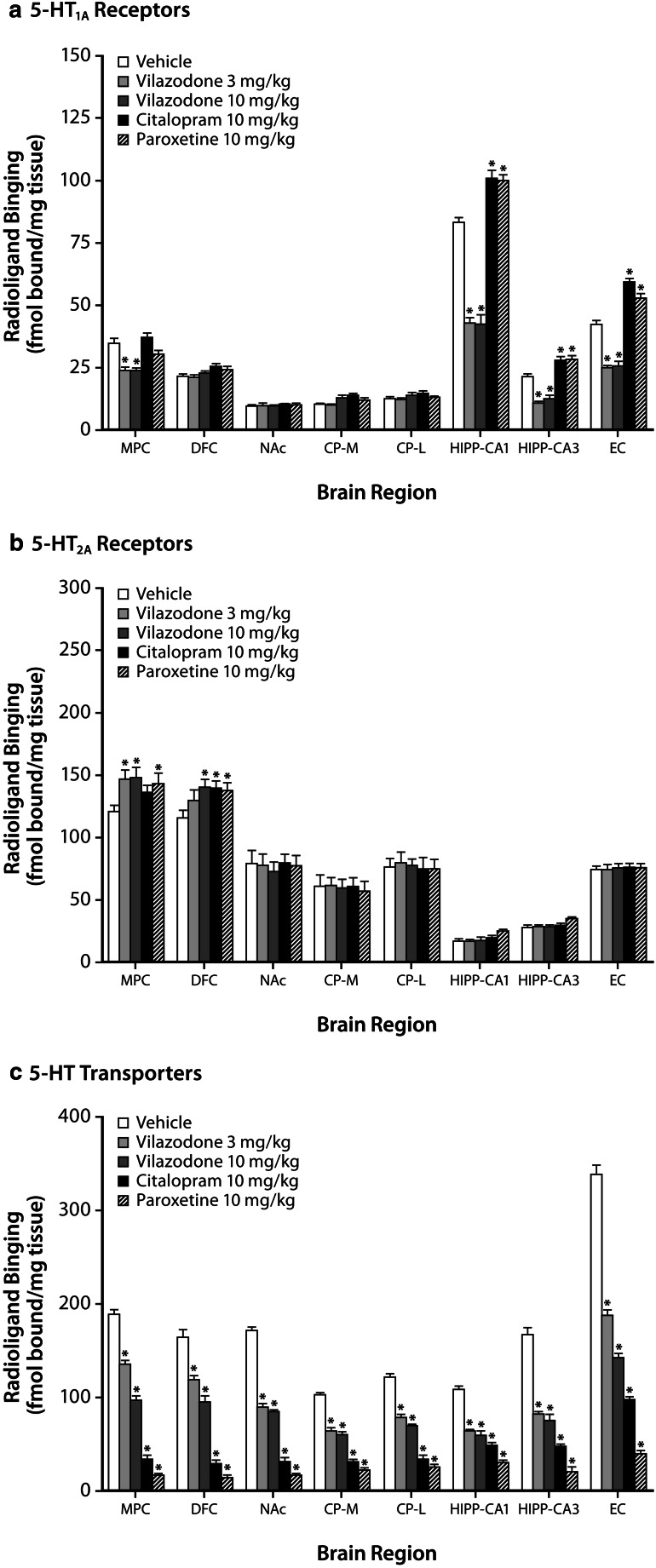
Effects of chronic (14 days) vilazodone, citalopram, and paroxetine treatment on the levels of serotonergic markers in rat forebrain regions. *CP-M* indicates caudate putamen-medial, *CP-L* caudate putamen-lateral, *DFC* dorsal frontal cortex, *EC* entorhinal cortex, *HIPP-CA*_*1*_ hippocampus CA_1_ region, *HIPP-CA*_*3*_ hippocampus CA_3_ region, *MPC* medial prefrontal cortex, *NAc* nucleus accumbens. Data are shown as the mean ± SEM (*n* = 8/group). *P < .05 vs vehicle (ANOVA, post hoc Dunnett *t* test)

In vilazodone-treated rats, 5-HT_1A_ receptor levels were reduced in the MPC, HIPP-CA_1_, HIPP-CA_3_, and EC regions (*P* < .05) (Fig. 4a, Table 1). In contrast, citalopram- and paroxetine-treated rats had increased 5-HT_1A_ receptor levels in the HIPP-CA_1_, HIPP-CA_3_, and EC regions (*P* < .05) (Fig. 4a, Table 1). Notably, vilazodone showed the opposite effects of citalopram or paroxetine on 5-HT_1A_ receptor levels in HIPP-CA_1_, HIPP-CA_3_, and EC brain regions.

**Table 1:** Mean percent changes from vehicle in serotonergic markers after chronic (14 days) vilazodone, citalopram, and paroxetine treatment

Brain region	Vilazodone (%)		Citalopram (%)	Paroxetine (%)
	3 mg/kg	10 mg/kg	10 mg/kg	10 mg/kg
5-HT_1A_				
MPC	−31*	−32*	NS	NS
HIPP-CA_1_	−49*	−49*	+21*	+20*
HIPP-CA_3_	−49*	−42*	+33*	+34*
EC	−42*	−42*	+39*	+24*
5-HT_2A_				
MPC	+22*	+23*	NS	+19*
DFC	NS	+21*	+21*	+19*
5-HT_T_				
MPC	−28*	−49*	−82*	−91*
DFC	−28*	−42*	−82*	−91*
NAc	−48*	−50*	−81*	−90*
CP-M	−37*	−41*	−69*	−78*
CP-L	−36*	−37*	−72*	−79*
HIPP-CA_1_	−41*	−45*	−54*	−72*
HIPP-CA_3_	−51*	−55*	−71*	−87*
EC	−45*	−58*	−71*	−88*

*CP-M* caudate putamen-medial, *CP-L* caudate putamen-lateral, *DFC* dorsal frontal cortex, *EC* entorhinal cortex, *HIPP-CA*_*1*_ hippocampus CA_1_ region, *HIPP-CA*_*3*_ hippocampus CA_3_ region, *MPC* medial prefrontal cortex, *NAc* nucleus accumbens, *NS* not significantly different from vehicle

*P < .05 versus vehicle (*n* = 8/group)

### 5-HT_2A_ receptor levels

Vilazodone-treated rats had increased 5-HT_2A_ receptor levels in the MPC and the DFC (10 mg/kg only) regions (*P* < .05) (Fig. 4b, Table 1). Similarly, paroxetine-treated rats had increased 5-HT_2A_ receptor levels in the MPC and DFC regions (*P* < .05) (Fig. 4b, Table 1). Citalopram-treated rats had increased 5-HT_2A_ receptor levels in the DFC region (*P* < .05) (Fig. 4b, Table 1).

### Serotonin transporter (5-HT_T_) levels

Chronic treatment with vilazodone, citalopram, and paroxetine reduced 5-HT_T_ levels in all brain regions (Fig. 4c, Table 1). Compared with vehicle, chronic vilazodone-treated (3 and 10 mg/kg/day) rats had dose-dependent reductions of 5-HT_T_ levels in the MPC, DFC, NAc, CPu, HIPP-CA_1_, HIPP-CA_3_, and EC regions (*P* < .05). Chronic citalopram- and paroxetine-treated rats (10 mg/kg/day for both) also had reductions in 5-HT_T_ levels compared to vehicle (*P* < .05); the magnitude of reductions in 5-HT_T_ levels was greater for citalopram- and paroxetine-treated rats than for rats treated with both doses of vilazodone (*P* < .05).

## Discussion

The current studies compared the effects of vilazodone, a combined SSRI and 5-HT_1A_ receptor partial agonist, and conventional SSRIs (citalopram and paroxetine) on male rat sexual behaviors and brain 5-HT receptor and transporter levels. SSRI treatment has been associated with dysfunction of the three sequential aspects of the sexual response cycle: sexual desire (i.e., libido), arousal (i.e., erectile function in men), and orgasm (Serretti and Chiesa 2009). Rats also exhibit a sexual response cycle, which includes introductory, copulatory, and ejaculatory phases, that is sensitive to SSRI treatment (Snoeren et al. 2014).

Vilazodone treatment had no adverse effects on copulatory and ejaculatory behaviors following acute, subchronic (7 days), and chronic (14 days) treatment; however, subchronic and chronic treatment with paroxetine, and to a lesser extent citalopram, inhibited copulatory efficiency and ejaculatory parameters (decreased frequency and increased latency to first ejaculation). The paroxetine and citalopram results were consistent with previous reports in rats (de Jong et al. 2005a, b; Waldinger et al. 2002) and similar to humans (Waldinger et al. 2001). Since the 5-HT system maintains an inhibitory tone on sexual behavior, chronic elevation of extracellular 5-HT by SSRI treatment is believed to underlie delayed and inhibited ejaculation in men (Rosen et al. 1999) and male rats (Chan et al. 2010). Notably, the acute treatments had no effects on sexual behavior which, given the inhibitory role of 5-HT in sexual behaviors, may seem surprising. Acute administration of SSRIs and vilazodone results in rapid and sharp increases in extracellular 5-HT levels in the rat medial and lateral cortices (van Amsterdam and Seyfried 2014) and in the ventral hippocampus (Page et al. 2002). The increase in 5-HT levels following vilazodone treatment was nearly two times greater than following citalopram, paroxetine, or fluoxetine (van Amsterdam and Seyfried 2014). However, evidence in humans and rats suggests that acute treatments of SSRIs generally do not cause sexual dysfunction (Olivier et al. 2011; Waldinger et al. 2001, 2002); rather, SSRI-related sexual dysfunction is likely dependent upon delayed neurochemical adaptions following chronic, but not acute, elevation of 5-HT as well as modulations of neuroendocrine and other neurotransmitter systems (Clayton et al. 2013; Olivier et al. 2011). One notable exception may be the SSRI, dapoxetine, which has been developed for the treatment of premature ejaculation. The acute effects of dapoxetine on ejaculatory function have been attributed to its pharmacokinetic profile (i.e., rapid absorption and elimination) and its targeting of excitatory thalamic and hypothalamic regions associated with ejaculatory response (Clément et al. 2012; McMahon 2011). However, although one preclinical study found high-dose dapoxetine to have limited effects on ejaculation latency in rapid-ejaculating rats, other studies in normal male rats did not show differences between dapoxetine and paroxetine (Olivier 2015). From this standpoint, dapoxetine does not appear to differ from other SSRIs in its pharmacologic ability to inhibit ejaculation latency in either rats or men (Olivier 2015). In contrast to vilazodone, acute administration of 5-HT_1A_ receptor agonists stimulates copulatory and ejaculatory behaviors in male rats (Arnone et al. 1995; de Jong et al. 2005b; Snoeren et al. 2014), which may be due to the differences between full and partial agonism at the 5-HT_1A_ receptor; other differences in acute neurophysiological and behavioral responses in rats have been reported between full 5-HT_1A_ receptor agonists and vilazodone (Bartoszyk et al. 1997; Page et al. 2002).

Since the impetus for this study was to better understand the adverse effects of SSRIs and other antidepressants on sexual functioning in patients who require such treatments, the current study focused on brain regions in the limbic system known to be associated with MDD and SSRI-mediated sexual dysfunction (Baldessarini 2006; Snoeren et al. 2014). However, it is important to note that other structures rich in 5-HT receptors, such as the raphé magnus, raphé pallidus, and gigantocelluar nuclei of the ventral medulla, may also contribute to the effects of antidepressants on male sexual behavior. Studies in rats have reported various effects of SSRIs on 5-HT receptor density. For example, repeated citalopram exposure was found to increase 5-HT_1A_ receptors and decrease 5-HT_2A_ receptors in the hippocampus in one study (Klimek et al. 1994), while a later study using positron emission tomography along with autoradiographic assays found no effect of chronic citalopram on 5-HT_1A_ receptor density in the dorsal raphé nucleus, frontal cortex, or hippocampus (Moulin-Sallanon et al. 2009). Repeated administration with paroxetine in rats was shown to have no effect on the density of 5-HT_1A_ receptors in the hypothalamus (Li et al. 1997). In the current study, chronic vilazodone treatment reduced postsynaptic 5-HT_1A_ receptor levels in the cortical and hippocampal regions, while citalopram and paroxetine increased 5-HT_1A_ receptor levels in the same brain regions. Importantly, the differential effects of the drugs on postsynaptic 5-HT_1A_ receptor levels occurred in the limbic cortex (MPC and EC) and the hippocampus. These are key brain substrates that receive afferent 5-HT projections from the dorsal and median raphé nucleus and are involved in the regulation of ejaculation (Snoeren et al. 2014). Stimulation of postsynaptic 5-HT_1A_ receptors by vilazodone via both direct activation and elevated extracellular 5-HT levels in the prefrontal cortex and hippocampus (Hughes et al. 2005; Page et al. 2002) may lead to 5-HT_1A_ receptor downregulation.

The results of 5-HT_1A_ receptor downregulation and lack of sexual side effects with vilazodone may complement a recent electrophysiology study by Ashby et al. This study reported that presynaptic 5-HT_1A_ autoreceptors in the dorsal raphé nuclei, which normally inhibit 5-HT neuron firing, showed much greater desensitization following chronic vilazodone treatment than following a similar treatment with fluoxetine (Ashby et al. 2013). Therefore, this desensitization would be expected to increase extracellular 5-HT release in afferent brain regions, including the cortex and hippocampus, and consequently result in a higher likelihood of sexual side effects following chronic vilazodone administration compared with conventional SSRIs. However, one possible explanation for the lack of sexual side effects of vilazodone is that the 5-HT_1A_ partial agonist activity of this molecule may stimulate the postsynaptic 5-HT_1A_ receptors which can counteract the inhibitory effects of elevated extracellular 5-HT resulting from presynaptic 5-HT_1A_ receptor desensitization and 5-HT_T_ blockade. In accordance with this explanation, systemic administration of several other 5-HT_1A_ receptor agonists and partial agonists enhanced ejaculatory behaviors in male rodents (Arnone et al. 1995; de Jong et al. 2005b; Snoeren et al. 2014). For instance, de Jong et al. (2005b) reported that a challenge with the full 5-HT_1A_ receptor agonist 8-OH-DPAT in male rats chronically treated with paroxetine restored the sexual behavior of these animals. Vilazodone has demonstrated comparable activity to 8-OH-DPAT at postsynaptic 5-HT_1A_ receptors in hippocampal tissue (Hughes et al. 2005). If this hypothesis is correct, the timing of the sexual behavior tests in rats relative to drug administration may be critical and will be investigated in a future study. In humans, however, this may be less of an issue because of the much longer half-life of vilazodone.

Chronic treatment with vilazodone, citalopram, and paroxetine reduced 5-HT_T_ levels in all of the tested forebrain regions. This is consistent with existing literature demonstrating that elevated extracellular 5-HT concentrations in vitro (Jorgensen et al. 2014) and chronic SSRI and serotonin norepinephrine reuptake inhibitor (SNRI) treatment in vivo (Mirza et al. 2007) can lead to reduced 5-HT_T_ expression. A consequence of reduced 5-HT_T_ availability is enhanced postsynaptic serotonergic neurotransmission, which may in turn contribute to sexual dysfunction (Chan et al. 2011). In the current study, the magnitude of reductions in 5-HT_T_ levels was greater for citalopram and paroxetine (maximum reductions of 80 and 91 %, respectively) than for vilazodone (maximum reduction of 58 %). Because of the differential effects of vilazodone relative to the SSRIs on both 5-HT_T_ and 5-HT_1A_ receptors, it is likely that the dynamics of extracellular 5-HT differ between rats that are chronically treated with the two different types of antidepressants.

The increases in cortical 5-HT_2A_ receptor levels following the different types of antidepressant treatments may suggest a common mechanism of action that mediates the beneficial therapeutic effects of dissimilar antidepressant drugs. The 5-HT_2A_ receptor is purported to have an inhibitory role in copulatory behavior, as shown by a decreased number of rats that initiated copulation after receiving 5-HT_2A_ receptor agonists in a sexual behavior test (Olivier et al. 2011). During the day 14 sexual behavior tests, the majority of citalopram- and paroxetine-treated rats had ≤2 ejaculations and several rats had no ejaculations. These results may be related to elevated 5-HT_2A_ receptor activity following increased expression in cortical areas.

## Conclusion

SSRI treatment is associated with direct negative effects on sexual function that may lead to treatment discontinuation and reduced quality of life (Hu et al. 2004). Combination therapy with SSRIs and 5-HT_1A_ agonists and partial agonists, such as buspirone, has been shown to ameliorate SSRI-induced sexual dysfunction (Clayton and Montejo 2006; Landen et al. 1999). In the current preclinical studies, unlike the conventional SSRIs, vilazodone was not associated with diminished copulatory or ejaculatory behaviors in male rats. These results support clinical trial data showing that vilazodone treatment, while effective in improving depressive symptoms, was associated with a relatively low incidence of direct adverse effects on sexual function (Clayton et al. 2013, 2015). The distinct effects of vilazodone on the 5-HT_T_ and 5-HT_1A_ receptors may enhance serotonergic transmission to achieve clinical efficacy in depression while limiting the adverse events of SSRI-induced sexual dysfunction.
